# Comparison of the new self-contained darkroom refractive screener versus table-top autorefractor and cycloplegia retinoscopy in detecting refractive error

**DOI:** 10.1186/s12886-023-03231-6

**Published:** 2023-11-27

**Authors:** Xianxian Wei, Lili Li, Li Jiang, Haiyan Lu, Huiyao Huang, Dedong Zhong, Liang Pan, Diefeng Wei, Yun Han, Hong Lin, Qi Chen

**Affiliations:** 1 Visual Science and Optometry Center, the People’s Hospital of Guangxi Zhuang Autonomous Region & Guangxi Key Laboratory of Eye Health & Guangxi Health Commission Key Laboratory of Ophthalmology and Related Systemic Diseases Artificial Intelligence Screening Technology & Institute of Ophthalmic Diseases, Guangxi Academy of Medical Sciences, Nanning, China; 2 Guilin Medical University, Guilin, China

**Keywords:** Refractive error, Refractive screener, Refractive screening, Autorefraction

## Abstract

**Purpose:**

By comparing the results of the new self-contained darkroom refractive screener (YD-SX-A) versus table-top autorefractor and cycloplegic retinoscopy, to evaluate the performance of the YD-SX-A in detecting refractive error in children and adolescents and then judge whether it can be used in refractive screening.

**Methods:**

Cross-sectional study. 1000 participants between the ages of 6 and 18 who visited the Optometry Center of the People's Hospital of Guangxi Zhuang Autonomous Region from June to December 2022 were selected. First, participants were instructed to measure their diopter with a table-top autorefractor (Topcon KR8800) and YD-SX-A in a noncycloplegic setting. After cycloplegia, they were retinoscopy by a professional optometrist. The results measured by three methods were collected respectively. To avoid deviation, only the right eye (1000 eyes) data were used in the statistical analysis. The Bland–Altman plots were used to evaluate the agreement of diopters measured by the three methods. The receiver operating characteristic (ROC) curves was used to analysis effectiveness of detecting refractive error of YD-SX-A.

**Results:**

The average age of participants was 10.77 ± 3.00 years, including 504 boys (50.4%) and 496 girls (49.6%). When YD-SX-A and cycloplegia retinoscopy (CR) were compared in the myopia group, there was no statistical difference in spherical equivalent (SE) (*P* > 0.05), but there was a statistical difference in diopter spherical (DS) and diopter cylinder (DC) (*P* < 0.05). Comparing the diopter results of Topcon KR8800 and CR, the difference between each test value in the myopia group was statistically significant (*P* < 0.05). In the hyperopia group, the comparison between YD-SX-A and CR showed no statistically significant differences in the DC (*P* > 0.05), but there were significant differences in the SE and DS (*P* < 0.05). In the astigmatism group, the SE, DS, and DC were statistically different, and the DC of YD-SX-A was lower than that of CR and Topcon KR8800. Bland–Altman plots indicated that YD-SX-A has a moderate agreement with CR and Topcon KR8800. The sensitivity and specificity of YD-SX-A for detecting myopia, hyperopia and astigmatism were 90.17% and 90.32%, 97.78% and 87.88%, 84.08% and 74.26%, respectively.

**Conclusion:**

This study has identified that YD-SX-A has shown good performance in both agreement and effectiveness in detecting refractive error when compared with Topcon KR8800 and CR. YD-SX-A could be a useful tool for large-scale population refractive screening.

## Background

Refractive error has become a global public health problem in recent years, with a growing incidence [1–3]. By 2050, there are expected to be 4.758 billion people with myopia around the world, or 49.5% of the worldwide population [4]. China is one of the countries with a high prevalence of myopia. According to the 2018 national myopia survey, the myopia rate among children and adolescents aged 6 to 18 in China is 53.6%, with approximately 100 million sufferers. Myopia not only affects daily activities, academic performance, and professional advancement but also causes amblyopia, fundus lesions, and even blindness when it develops into high myopia [5–8]. The continued growth of the myopia population will have an impact on both social selection and economic development [9]. Therefore, extensive vision screening is an important task for the whole society, and the medical and health institutions play a key role in this respect [10–15]. Only through a large-scale refractive screening can we find high-risk children in advance, and primary prevention measures such as increasing outdoor time can be implemented timely [16–19]. Early screening of myopia children and subsequent clinical intervention with low concentration atropine, orthokeratology lenses, and other secondary preventive measures can effectively slow down the progression of myopia and lower the incidence of high myopia [20–23].

Cycloplegia retinoscopy (CR) is considered to be the gold standard method for measuring refractive error [24, 25], which inhibits ciliary muscle accommodation and detects real refractive error. However, there are some limitations to CR, including the fact that the operators must receive professional and technical clinical ophthalmology training, and patients may experience a number of temporary side effects, such as blurred vision and photophobia [26, 27]. Cycloplegic drops like tropicamide take 30–40 min to take effect, and parents and kids need longer or additional appointment times [28], so the method of CR is not suitable for large-scale screening. Autorefractors, including hand-held and table-top autorefractors, play an essential role in detecting refractive error through screening [29]. Previous studies have demonstrated that the majority of current autorefractors are reliable and accurate compared to subjective refraction [30, 31]. Although these devices may rapidly test and report refractive errors, they are heavy, non-portable, and easily affected by the accommodation of children and adolescents, the operation technology of the examiner, and the cooperation of the examinee [27].

At present, the common refractive screening instruments have the advantages of compactness, good portability, and fast measurement speed, such as the Welch Allyn SureSight Vision Screener, PlusoptiX Photoscreener, Retinomax Autorefractor, Spot Photoscreener, and more [32–36]. Even though numerous studies have found that most of them can be used for refractive screening, their accuracy is easily affected by the accommodation of children's eyes and the influence of the detection environment and distance [37, 38]. China has a sizable population but a relative lack of medical personnel and supplies. Automatic screening equipment can effectively improve the effectiveness of eye care [14]. Therefore, it is great significance to further improve the screening performance and detection efficiency of refractive screening instruments.

The new self-contained darkroom refractive screener (YD-SX-A, Guangxi Nanning Gardener Medical Instrument Co., Ltd., China) is a binocular photoscreener with built-in 1 m long cylindrical darkroom. The upper part is shaped to fit the features of a human face, while the lower portion includes a foundation that can be securely put on the ground. When it is time to move, it may be folded and placed in a trunk or backpack. Because it is battery powered, that is no need to plug it in when using it. The operation interface and test results can be displayed on the screen of an external tablet or mobile phone that is linked through WiFi and has the instrument-specific APP downloaded beforehand. The examinees need simply come close to the upper detection place, fully open their eyes during the detection to look at the infrared emission location at the bottom, and the binocular diopter can be measured in 2 to 5 s. The instrument will automatically detect three times and then display the average diopter value. The YD-SX-A’s dark room causes the pupil to enlarge during the detection of refractive error, and it used an infrared camera to take and analyze pictures of the red pupil reflex in order to assess the alignment of both eyes and calculate the refractive error. The diopter results are acquired by automatically measuring three times and obtaining the average value. It is similar with some photoscreener such as PlusoptiX Photoscreener, Spot Photoscreener, but the difference is that YD-SX-A has a fixed detection distance and dark room environment [32]. The YD-SX-A has the advantages of simple operation, convenience and cooperation, making it practical and appropriate for infants and children who find it difficult to cooperate with table-top autorefractors.

The purpose of this study was to compare refractive measurements taken in children and adolescents using the YD-SX-A, Topcon KR8800 and CR to evaluate the performance of the YD-SX-A in detecting refractive error.

## Methods

### Participants

A total of 1000 participants between the ages of 6 and 18 who visited the Optometry Center of the People's Hospital of Guangxi Zhuang Autonomous Region from June to December 2022 were selected. The ophthalmologist evaluated the anterior segment and examined the lens, vitreous, and fundus with a slit lamp and indirect ophthalmoscopic to confirm that participants had no other eye diseases. The parents or guardians of all participants have signed an informed consent. The study was reviewed and approved by the Ethics Committee of the Guangxi Zhuang Autonomous Region People's Hospital (NO: KY-ZC-2022–135) and strictly followed the Declaration of Helsinki.

### Check steps

First, participants were instructed to measure their diopter using a table-mounted autorefractor (Topcon KR8800) and the YD-SX-A without cycloplegia. And then, if the children and adolescents are considered eligible for cycloplegia, they will receive intraocular pressure measurement with written informed consent and then pupil dilation. As a safe cycloplegic refraction agent, tropicamide, a synthetic analogue of tropic acid, is known to restore near vision more quickly and cause less stinging than cyclopentolate [26, 39]. Many recent studies have suggested that tropicamide can be used in cycloplegic as an alternative of cyclopentolate [40]. In our research, one drop of topical tropicamide 0.5% was applied to each eye five minutes apart to induce cycloplegia. Pupil size and light reflex were examined at 45 min after administration of the first drop of tropicamide, and if the pupil was dilated to 6 mm and the light reflex was absent, then cycloplegia was considered established. An experienced optician performed a retinoscopy on them after that. The results measured by three methods were collected. To avoid deviation, only the right eye (1000 eyes) data were used in statistical analysis [41].

### Statistical analysis

A database was created using Epidata 3.1, then all data were entered by the same person and processed using IBM SPSS 24.0. The refraction data includes diopter spherical (DS), diopter cylinder (DC), and axis (a). The spherical equivalent (SE) was calculated according to the following formula: SE = S + C/2. Myopia was defined as SE < -0.5D, hyperopia as SE >  + 2.0D, and astigmatism as DC < -0.75D. Since the data does not follow a normal distribution, expressed by interquartile range [D, *M* (P25, P75)]. The Wilcoxon signed-rank test was used to compare the differences between the diopters obtained by YD-SX-A and Topcon KR8800 versus CR in different groups. The Bland–Altman plots were used to evaluate the agreement of diopters measured by the three methods. Taking the results of CR as the gold standard, the sensitivity and specificity of myopia, hyperopia, and astigmatism are determined by the results of YD-SX-A and Topcon KR8800. The receiver operating characteristic (ROC) curve was employed to select the best cutoff points related to the appropriate sensitivity and specificity of YD-SX-A and Topcon KR8800 to detect refractive error and then compare the results of the above two methods. The linear regression analysis was used to evaluate the quantitative relationship between the results of YD-SX-A and CR. Statistical significance was defined as *P* < 0.05.

## Results

### General characteristics

**Fig. 1:**
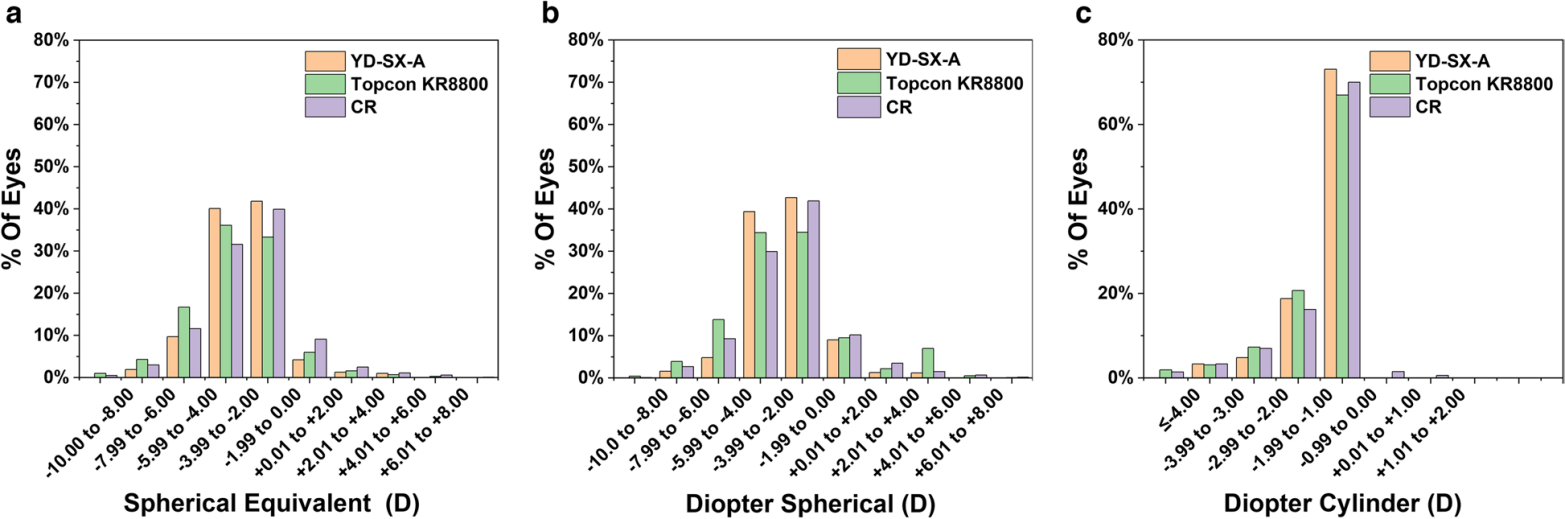
Histogram illustrating the distribution of refractive error in diopter. SE (**a**); DS (**b**); DC (**c**)

A total of 1000 participants aged 6 to 18 years were included in this study, with an average age of 10.77 ± 3.00 years, including 504 boys (50.4%) and 496 girls (49.6%). There were no statistical differences in gender distribution by χ2 test (*P* = 0.390). 783 participants (78.3%) had myopia as defined according to the results of the CR. Meanwhile, 45 participants (4.5%) had hyperopia, and 289 participants (28.9%) had astigmatism. The distribution of diopter is shown in Fig. 1.

#### Comparison of results

According to the analysis of the total number of data, the difference in SE, DS, and DC between YD-SX-A or Topcon KR8800 and CR were statistically significant respectively (*P* < 0.05). When YD-SX-A and CR were compared in the myopia group, there was no statistical difference in SE (*P* > 0.05), but there was a statistical difference in DS and DC (*P* < 0.05). Comparing the diopter results of Topcon KR8800 and CR, the difference between each test value in the myopia group was statistically significant (*P* < 0.05). The SE of YD-SX-A was more incline to myopia than that of CR, but is more incline to orthoptic than that of Topcon KR8800 in the myopia group. In the hyperopia group, the comparison between YD-SX-A and CR showed no statistically significant differences in the DC (*P* > 0.05), but there were significant differences in the SE and DS (*P* < 0.05). The SE of YD-SX-A was more inclined to orthoptic than those of CR and Topcon KR8800 in the hyperopia group. In the astigmatism group, the SE, DS, and DC were statistically different, and the DC of YD-SX-A was lower than that of CR and Topcon KR8800. Table 1 presents the data distribution of YD-SX-A, Topcon KR8800 and CR in different groups.

**Table 1:** Data distribution of YD-SX-A, Topcon KR8800 and CR in different groups [D, *M* (*P*_25_, *P*_75_)]

**Method**	**Diopter of all participants (1000 eyes)**			**Diopter of myopic group (783 eyes)**		
	**SE(D)**	**DS (D)**	**DC(D)**	**SE(D)**	**DS (D)**	**DC(D)**
YD-SX-A	-2.06(-3.22, -1.00) *P* < 0.001*	-1.50(-2.75, -0.50) *P* < 0.001*	-0.50(-1.00, -0.25) *P* = 0.032*	-2.50(-3.38, -1.50) *P* = 0.729	-2.25(-3.00, -1.25) *P* = 0.02*	-0.50(-0.75, -0.25) *P* = 0.002*
Topcon KR8800	-2.38(-3.75, -1.25) *P* < 0.001*	-2.*00(-3.25, -0.75)* *P* < 0.001*	-0.50(-1.25, -0.25) *P* < 0.001*	-2.88(-4.13, -1.88) *P* < 0.001*	-2.50(-3.75, -1.50) *P* < 0.001*	-0.50(-1.00, -0.25) *P* < 0.001*
CR	-1.80(-3.30, -0.80)	-1.50(-2.75, -0.50)	-0.50(-1.00, 0.00)	-2.30(-3.50, -1.50)	-2.00(-3.25, -1.25)	-0.50(-0.75, 0.00)
**Method**	**Diopter of hyperopia group (45 eyes)**			**Diopter of astigmatism group (289 eyes)**		
	**SE(D)**	**DS (D)**	**DC(D)**	**SE(D)**	**DS (D)**	**DC(D)**
YD-SX-A	0.25(-0.38, 0.94) *P* < 0.001*	0.75(0.25, 1.50) *P* < 0.001*	-0.75(-1.50, -0.50) *P* = 0.159	-1.63(-3.38, -0.69) *P* < 0.001*	-0.50(-2.37, 0.37) *P* < 0.001*	-2.00(-2.75, -1.37) *P* < 0.001*
Topcon KR8800	0.75(-0.06, 2.13) *P* < 0.001*	1.50(0.75, 3.00) *P* < 0.001*	-1.25(-2.12, -0.50) *P* < 0.001*	-1.75(-3.94, 0.38) *P* < 0.001*	-0.25(-2.87, 1.12) *P* < 0.001*	-2.50(-3.25, -2.00) *P* < 0.001*
CR	1.90(1.10, 3.45)	2.50(1.75, 4.12)	-1.00(-2.00, -0.25)	-1.10(-3.60, 0.45)	0.50(-2.25, 2.00)	-2.50(-3.25, -2.00)

^*^Comparison with CR:* P* < 0.05

### Agreement analysis

**Fig. 2:**
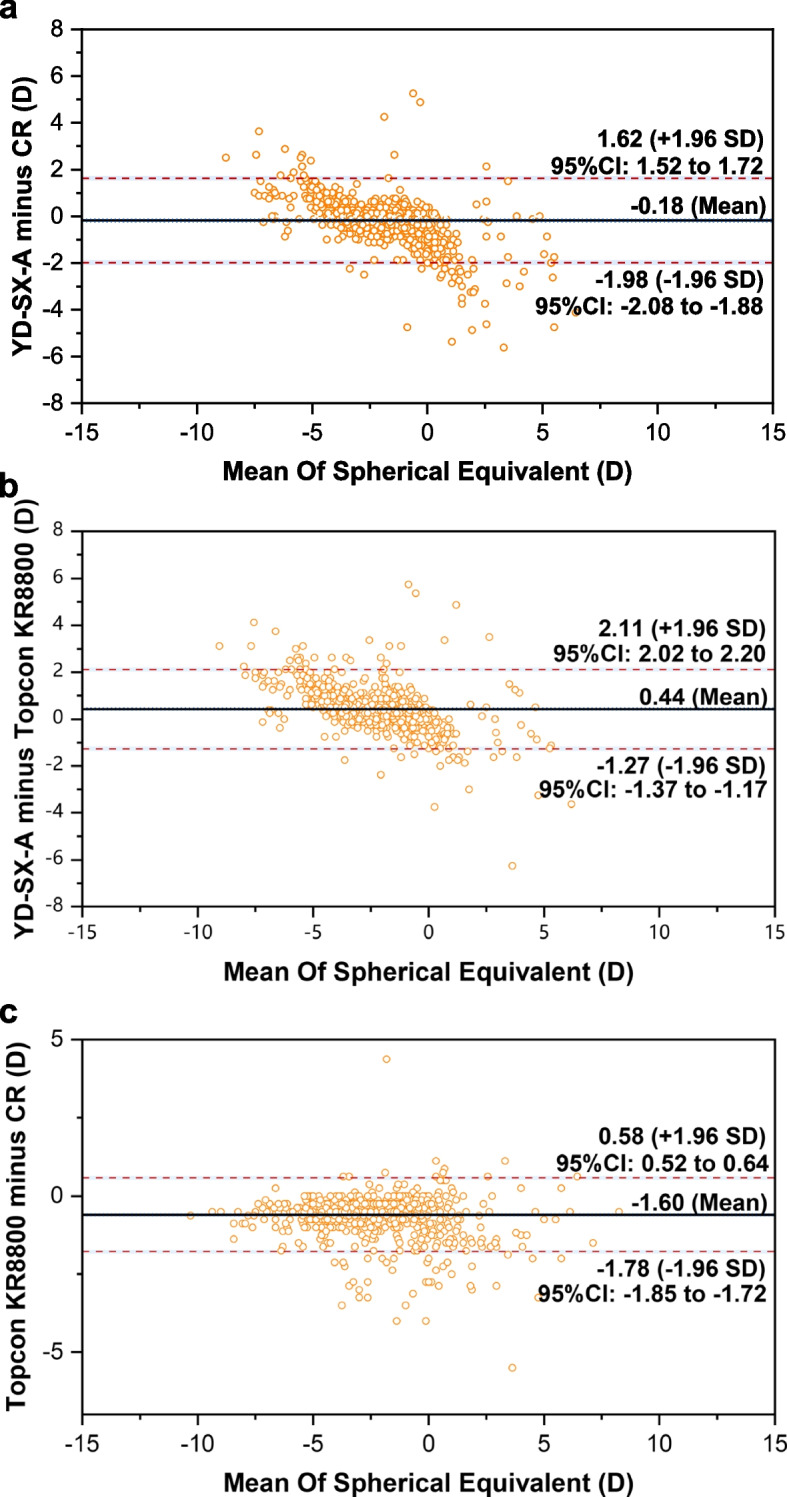
Bland–Altman plots of the difference in SE between YD-SX-A and CR (**a**), YD-SX-A and Topcon KR8800 (**b**), Topcon KR8800 and CR (**c**)

**Fig. 3:**
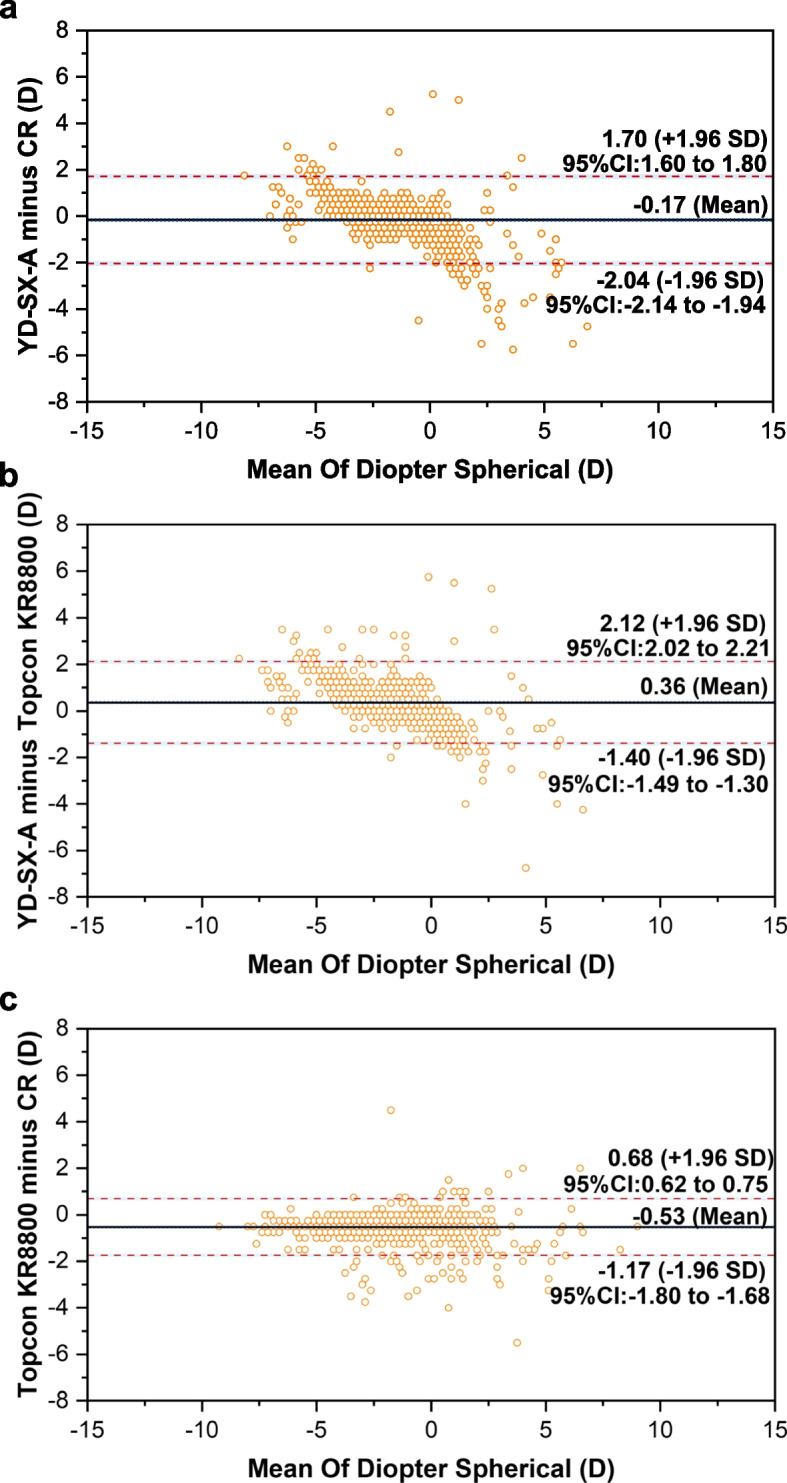
Bland–Altman plots of the difference in DS between YD-SX-A and CR (**a**), YD-SX-A and Topcon KR8800 (**b**), Topcon KR8800 and CR (**c**)

**Fig. 4:**
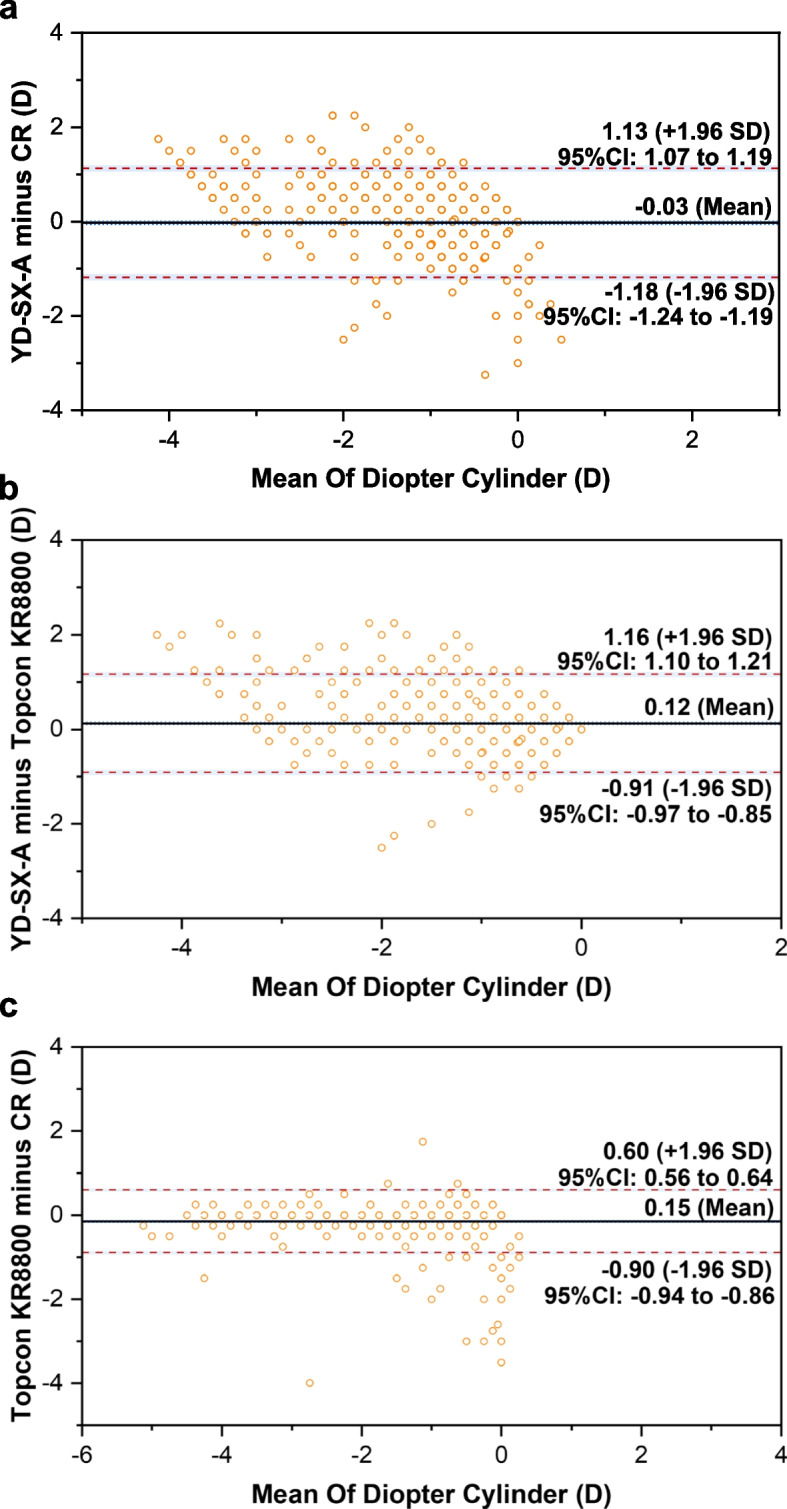
Bland–Altman plots of the difference in DC between YD-SX-A and CR (**a**), YD-SX-A and Topcon KR8800 (**b**), and Topcon KR8800 and CR (**c**)

The Bland–Altman plots were used to evaluate the agreement of diopters measured by the three methods. As demonstrated in Table 2, the mean differences and 95% limits of agreement in SE between YD-SX-A and CR, YD-SX-A and Topcon KR8800, Topcon KR8800 and CR were -0.18D (95% limits of agreement -1.98 to 1.62), 0.44D (95% limits of agreement -1.27 to 2.11), -1.60D (95% limits of agreement t -1.78 to 0.58), respectively (Fig. 2). In addition, for diopter spherical, the mean differences and 95% limits of agreement were -0.17D (95% limits of agreement -2.04 to 1.70), 0.36D (95% limits of agreement -1.40 to 2.12), -0.53D (95% limits of agreement -1.17 to 0.68) (Fig. 3). For diopter cylinder, the mean differences and 95% limits of agreement were -0.03D (95% limits of agreement -1.18 to 1.13), 0.12D (95% limits of agreement -0.91to 1.16), 0.15D (95% limits of agreement -0.90 to 0.60) (Fig. 4).

**Table 2:** Agreement in mean refractive components between three methods

	SE	DS	DC
YD-SX-A minus CR			
Mean of difference	-0.18	-0.17	-0.03
95%LOA	-1.98 to 1.62	-2.04 to 1.70	-1.18 to 1.13
Number within the 95% LOA	841(84.1%)	966(96.6%)	951(95.1%)
YD-SX-A minus Topcon KR8800			
Mean of difference	0.44	0.36	0.12
95%LOA	-1.27 to 2.11	-1.40 to 2.12	-0.91to 1.16
Number within the 95% LOA	945(94.5%)	947(94.7%)	934(93.4%)
Topcon KR8800 minus CR			
Mean of difference	-1.60	-0.53	0.15
95%LOA	-1.78 to 0.58	-1.17 to 0.68	-0.90 to 0.60
Number within the 95% LOA	940(94.0%)	945(94.5%)	971(97.1%)

**Fig. 5:**
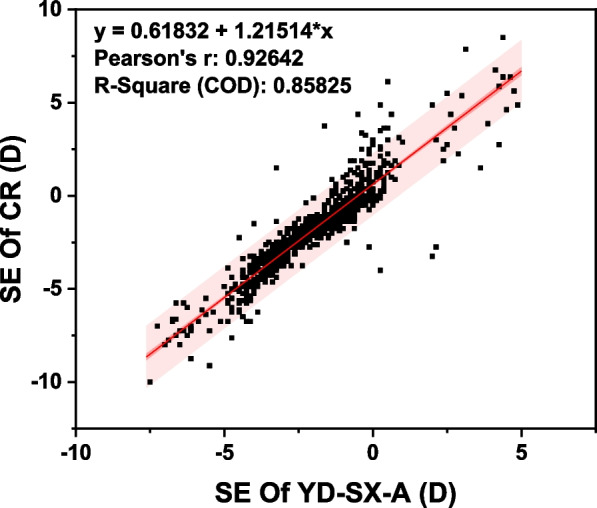
The correlation of SE between YD-SX-A and CR

Regression was used to evaluate the quantitative relationship between the results of YD-SX-A and CR. A linear regression model (*r* = 0.9261, *P* < 0.001) indicated a strong linear correlation. It was shown in Fig. 5.

### Accuracy evaluation

**Fig. 6:**
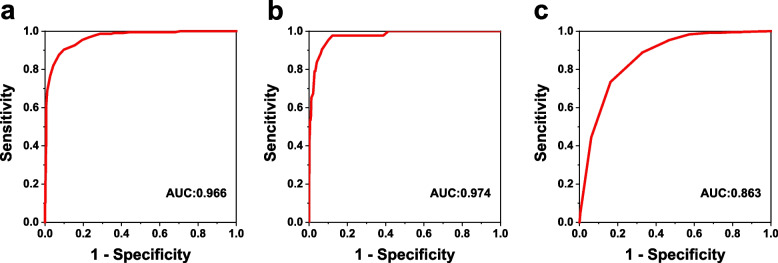
ROC curves for detecting refractive error in YD-SX-A. Myopia (**a**); Hyperopia (**b**); Astigmatism (**c**)

**Fig. 7:**
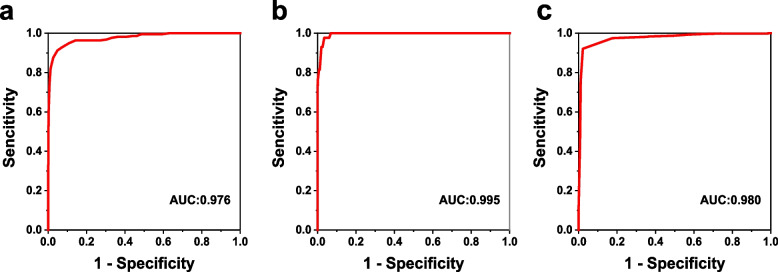
ROC curves for detecting refractive error in Topcon KR8800. Myopia (**a**); Hyperopia (**b**); Astigmatism (**c**)

The sensitivity and specificity of YD-SX-A and Topcon KR8800 in detecting refractive error are shown in Table 3. With CR as the gold standard, the area under the curve (AUC) determined by YD-SX-A as myopia was 0.966, and the best cutoff value was -1.063D; the sensitivity and specificity were 90.17% and 90.32% at this cutoff value. The AUC and cutoff determined as hyperopia were 0.975 and -0.562D, and the sensitivity and specificity were 97.78% and 87.88% at this cutoff. The AUC and cutoff determined as astigmatism were 0.866 and -0.725D, and the sensitivity and specificity were 84.08% and 74.26% at this cutoff. The ROC curve in Fig. 6 was used to assess the effectiveness of YD-SX-A in detecting refractive error. In addition, the sensitivity range of Topcon KR8800 to determine ametropia is 95.15% to 97.58%, the specificity range is 91.24% to 96.55%, and the cutoff range is -1.188 to 0.312. The ROC curve in Fig. 7 was used to assess the effectiveness of Topcon KR8800 in detecting refractive error.

**Table 3:** AUC, sensitivity and specificity to detect refractive error with YD-SX-A and Topcon KR8800cutoff values derived from ROC curves

WHO criteria	Myopia(SE < -0.5D)	Hyperopia(SE > + 2.0D)	Astigmatism(DC < -0.75D)
**YD-SX-A**			
AUC	0.966	0.974	0.863
Cutoff	-1.063	-0.562	-0.725
Sensitivity	90.17%	97.78%	84.08%
Specificity	90.32%	87.88%	74.26%
**Topcon KR8800**			
AUC	0.976	0.995	0.980
Cutoff	-1.188	0.312	-0.875
Sensitivity	95.15%	97.78%	97.58%
Specificity	91.24%	96.55%	93.25%

## Discussion

In order to prevent children and adolescents from suffering from refractive amblyopia, strabismus, cataract, glaucoma, and myopia fundus disease [5–8], refractive screening project was initiated nationwide to monitor the prevalence of myopia among children in China. Many studies have been conducted in the past to compare various refractive screening instruments, and most researchers agree that those instruments are appropriate for use [32–34]. In this study we compared the refractive error estimates of YD-SX-A to Topcon KR8800 and CR to evaluate the performance of the YD-SX-A in detecting refractive error.

In accordance with the present results, we can find that compared with the results of CR, YD-SX-A tended to overestimate myopia, underestimate hyperopia and astigmatism. This tendency was also found in other screeners, such as PlusoptiX S08 and Retinomax K-Plus2 [32]. In contrast to these devices, the YD-SX-A has a fixed detection distance and a dark room environment, which reduces the influence of detection distance and environment on the results. In the myopia group, the SE of YD-SX-A was closer to CR than Topcon KR8800, indicating that YD-SX-A underestimates myopia when compared to Topcon KR8800. This can be explained by the decrease accommodative response due to the dark room of YD-SX-A, thereby minimizing non-cycloplegic effects. In the astigmatism group, we mainly analyzed the comparison of DC and found that YD-SX-A underestimated astigmatism, and the comparisons with Topcon KR8800 and CR were statistically significant. The possible reason for this is that YD-SX-A cannot detect corneal curvature, resulting in a significant difference between the DC measured by YD-SX-A and the DC measured by Topcon KR8800 and CR. The performance could be further improved by adjusting referral criteria based on ROC analysis. This also raises the question of whether the accuracy can be improved by combining the algorithm and clinical data by increasing the database.

The Bland–Altman plots showed moderate agreement between YD-SX-A and CR, except that the coverage rate of SE is 84.1% and the DS and DC value exceed 90%. In addition, the coverage rate of each test value of YD-SX-A and Topcon KR8800 is about 95.0%. Therefore, YD-SX-A is more consistent with Topcon KR8800 than CR. This may be due to the fact that it is still affected by accommodation when detecting refractive error. However, it also demonstrates that YD-SX-A can be used in the refractive screening instead of the autorefractors.

Both the YD-SX-A and Topcon KR8800 have high sensitivity and specificity in detecting myopia (all values are greater than 90%). For detecting hyperopia, YD-SX-A had a lower specificity than Topcon KR8800. On the other hand, YD-SX-A showed lower sensitivity and specificity of astigmatism. However, considering that YD-SX-A provides relatively higher sensitivity (90.17% in myopia and 97.78% in hyperopia), it gives a reliable performance in detecting general refractive error. All results are shown in Table 2.

Of course, YD-SX-A also has some limitations. When detecting strabismus, high myopia, and high hyperopia, the results can be error prone, as a result, detections take more time, even the diopter cannot be measured. This is due to the device is a binocular refraction screener, which means that when the examinee has obvious strabismus, it is unable to acquire the refractive information for both eyes at the same time. But we can exclude and early detect strabismus patients through the function of capturing images, just like other photoscreeners [35, 42]. The measurement range of YD-SX-A is -7.50D to + 7.50D sphere/cylinder (0.25 increments), and axis 0° to 180° (1° increments). It will display < -7.50D or >  + 7.50D when the examinee has high myopia or high hyperopia. In these respects, YD-SX-A is inferior to the autorefractors.

The study still has limitations. First, the majority of those who got examinations at the Optometry Center of the People's Hospital of Guangxi Zhuang Autonomous Region had ametropia, so they were unable to accurately represent the distribution of diopters at the screening location. Second, there was no test on diopter after cycloplegic YD-SX-A, and no comparison was made on the difference in diopter before and after cycloplegia to understand the impact of ciliary muscle paralysis on YD-SX-A. But we analyzed the data before and after cycloplegia with a small sample, and the results reveal that there wasn’t much of a difference between the two. In future studies, we can further evaluate the accuracy of the instrument by expanding the sample size, including adding preschool children, and field screening data.

## Conclusions

YD-SX-A has shown a good performance in both agreement and effectiveness in detecting refractive error. It can be applied to schools and operated by school doctors and other personnel after simple training because of its simple and convenient operation. It will greatly solve the problem that refraction screening is difficult to carry out comprehensively due to insufficient professional optometry personnel and equipment. These findings indicated that YD-SX-A might be an effective instrument for large-scale population refractive screening.

## Data Availability

The data that support the findings of this study are available from the corresponding author upon reasonable request.
